# Informed consent practices for exome sequencing: An interview study with clinical geneticists in the Netherlands

**DOI:** 10.1002/mgg3.1882

**Published:** 2022-02-11

**Authors:** Wendy Bos, Eline M. Bunnik

**Affiliations:** ^1^ Department of Medical Ethics, Philosophy and History of Medicine Erasmus MC, University Medical Centre Rotterdam Rotterdam the Netherlands

**Keywords:** exome sequencing, genetic counseling, informed consent, interview study, mainstreaming

## Abstract

**Background:**

Genomic sequencing is being used more frequently in the clinic, not only by clinical geneticists, but also by other specialists (“mainstreaming”). The use of genomic sequencing gives rise to challenges regarding informed consent, as it can yield more, and more complex results.

**Methods:**

This study maps the informed consent process for exome sequencing in the Netherlands by means of semistructured interviews with 14 clinical geneticists. Interviewees were asked about their strategies for informing patients about exome sequencing and supporting patients in their decision making, about what they think of as essential information elements, about the challenges they experience, and about their preferences for future policy and practice.

**Results:**

Clinical geneticists typically discuss the following topics: the nature and aim of the test, the possible results (including unsolicited or incidental findings and Variants of Uncertain Significance) of the test and the consequences of those results for the patient and their family members. Some clinical geneticists use a layered approach to informed consent, meaning that they give short and concise information at first, and provide more detailed information depending on the situation or the needs of the patient.

**Conclusion:**

During pre‐test counseling for genomic sequencing, clinical geneticists use various strategies to enhance patient understanding and personalization of the informed consent process. Going forward, layering information may be part of a solution to ethical challenges of informed consent, also in mainstream settings.

## Abstract 2

The use of clinical genomic sequencing gives rise to challenges regarding informed consent, as it can yield more, and more complex results. This study maps the informed consent process for exome sequencing in the Netherlands by means of semistructured interviews with clinical geneticists. It contains lessons for clinicians for informed consent for exome sequencing in practice, in both clinical genetics and mainstream settings.

## 1 INTRODUCTION

Genomic sequencing tests are used frequently for diagnostic purposes in clinical settings. In using exome sequencing or gene panels, the exome is analyzed either in full or with a primary focus on a set of genes (i.e., gene panel) known to be associated with the disease with which a patient presents to the clinic. The clinical use of exome sequencing or gene panels is met with various ethical challenges, notably in relation to informed consent (Burke et al., 2013; Manolio et al., 2013; Vrijenhoek et al., 2015). Exome sequencing or gene panels are complex tests that tend to generate large quantities of genomic data, including findings that are beyond the purpose of the test (unsolicited or incidental findings) and unclear results or variants of uncertain significance (VUS). How can adequate informed consent be guaranteed?

As genomic sequencing as a diagnostic tool is expected to be used on a larger scale in the future, and also outside of the field of clinical genetics, there are further challenges to be expected. Upscaling of genomic sequencing will put more pressure on clinical genetics departments. Also, it is expected that other specialists (i.e., non‐geneticist clinicians) will initiate genomic sequencing tests themselves more often, a trend that is referred to as “mainstreaming” (Kemp et al., 2019; McLeavy et al., 2020; Rahman, 2014). Oncologists, cardiologists, neurologists, psychiatrists, pediatricians, and other non‐geneticist clinicians are increasingly ordering genomic tests directly. In mainstream settings, time for counseling and informed consent will be scarce. For that reason, there is need for efficient and goal‐oriented informed consent. As the trend toward more genomic sequencing in the clinic is already ongoing, it is important to first assess current informed consent practices for genomic sequencing. In clinical genetics, after all, pre‐test counseling has been conducted for decades, and the profession is characterized by a strong tradition of ethical thinking and practices of informed consent. How are clinical geneticists currently providing information to patients about genomic sequencing?

The primary aim of informed consent is to foster autonomous decision‐making in patients. This relates to the ethical principle of respect for autonomy of patients (Faden & Beauchamp, 1986). A second aim is to prepare patients for the procedure and to help them understand relevant information about the test, the test results and the consequences thereof. Test results can be impactful and psychologically harmful to patients and their family members. Proper preparation can help prevent such harm. This relates to the ethical principle of non‐maleficence (Manson & O'Neill, 2007).

Voluntariness, competence, and “informedness” are three conditions for informed consent (Thompson & McNamee, 2017). The latter entails that proper and understandable information must be provided or “disclosed.” Understandability is one of the bigger challenges for informed consent for genomic sequencing; as more genes are being sequenced and analyzed, the variety of possible results and their possible consequences increases. Genomic sequencing yields a lot of information, but also complicated information, for patients to process (Tomlinson et al., 2016). For doctors, it is likely not possible to disclose all that information to patients. Ethically, it might not even be necessary to do so, as more information is not always better information. In the theoretical literature on informed consent for genomic testing in other than clinical settings, the idea of tailored or personalized consent has been proposed to improve understandability and autonomous decision‐making (Bunnik, Janssens, et al., 2013; Bunnik, de Jong, et al., 2013). Are clinical geneticists bringing theoretical approaches to practice? And how do these translate to the mainstream setting?

Ever since exome sequencing has found its way to the clinic, only a few empirical studies have been done on informed consent for exome sequencing in the clinical setting, mostly with a focus on unsolicited findings, that is, findings of relevance to the health of the patients but beyond the purpose of the test (Bergner et al., 2014; Rigter et al., 2014). Bernhardt et al. conducted an interview study on the process and content of informed consent for genomic sequencing in 2015, and suggested that informed consent practices should focus on key issues that may be misunderstood by patients (Bernhardt et al., 2015). A survey and focus group study conducted by Gore et al. in 2017 found that North American genetic counselors consider “engaged decision‐making,” “assessing understanding,” and “managing expectations” most important “elements” of obtaining informed consent (Gore et al., 2019). When these studies were undertaken, the level of experience with genomic sequencing among respondents was variable. As genomic sequencing is now being used more widely in the clinic, it is worthwhile to investigate informed consent practices again, as clinical geneticists are more experienced. A recent interview study conducted in Europe, Australia, and Canada reported concerns among genetic health professionals about the vast amount of information to be conveyed during informed consent sessions for genomic sequencing (Vears et al., 2020). As these empirical studies do not offer in‐depth insight in the strategies that doctors use for making information understandable and for personalizing information, the current study aims to fill this gap.

In many countries, informed consent is not only a moral requirement, but also a legal one. In the Netherlands, this is regulated in the Dutch Medical Treatment Agreement Act of 1994 (Wet op de geneeskundige behandelingsovereenkomst (WGBO) [Medical Treatment Agreement Act], 1994). This act governs the rights and duties of patients and health care professionals regarding medical treatment, including informed consent. It requires that healthcare professionals inform patients about “what they reasonably need to know” about the following aspects: a. the nature and aim of the procedure, b. the expected consequences and risks of the procedure for the health of the patient, c. alternative procedures and alternative practitioners, d. the state and prospects of the patient's health related to the possible procedures, and e. when the procedure may take place and the duration of the procedure. In January 2020, the act was adjusted, and it was added that the doctor must engage the patient in a dialog and explicitly invite the patient to ask questions. Also, the doctor must inform the patient about the option of not taking the treatment or test. This adjustment of the act aimed at ensuring that “shared decision‐making” is now a legal requirement of the doctor–patient relationship (Ubbink et al., 2021). Dutch clinical geneticists must also comply with the guideline concerning counseling for genomic sequencing issued by the Dutch Association of Clinical Genetics in 2016 (Vereniging voor Klinische Genetica Nederland (VKGN) [Dutch Association of Clinical Genetics]; Richtlijn Counseling bij genoombrede detectie van chromosoomveranderingen (CNV) middels array of NGS diagnostiek, 2016). This guideline recommends counselors to at least discuss the following topics with patients who are offered genomic sequencing: the aim and method of the test, the possible outcomes (including unsolicited findings) and their consequences for the further treatment of the patient, possible consequences for the health of the patient and his or her family members, when and how the results are communicated and the possibility to choose not to take the test. In practice, however, each clinical geneticist may have his or her own personal approach or style when counseling patients and asking for informed consent.

The aim of this study was to map current informed consent practices for exome sequencing in the Netherlands. To this end, we conducted interviews with clinical geneticists about practices of informed consent for exome sequencing of competent adult patients. Competent adults were chosen as they are the paradigm subjects for informed consent, that is, persons who make decisions for themselves. Proxy consent for children or incompetent adults gives rise to additional ethical questions, which could distract from the focus on the process and content elements of informed consent itself. Interviews were chosen as a research method as they offer the opportunity for clinical geneticists to explain and reflect on the strategies they use during the informed consent process, as well as on any challenges they experience.

## 2 MATERIALS AND METHODS

### 2.1 Interviews

Semistructured interviews were held with clinical geneticists to study the process and content of the pre‐test counseling for genomic sequencing and clinicians' strategies for informing patients and their views on the informational needs of patients. The interviews were conducted in accordance with the COREQ checklist (see Supporting Information) (Tong et al., 2007). The research questions were (i) how do clinical geneticists inform competent adult patients about exome sequencing?; (ii) what difficulties and challenges do they experience? And (iii) what are their recommendations and wishes for future policy and practice?

### 2.2 Research participants

Clinical geneticists were selected from six out of eight academic hospitals in the Netherlands, based on their experience with counseling competent adult patients for exome sequencing and/or larger gene panels. Sampling was done by a combination of purposive sampling and snowball sampling. Respondents were recruited through a request by email to the heads of the clinical genetics departments. Diversity in genetic subspecialism, age and years of experience as a clinical geneticist was sought during recruitment. Potential participants received a short information letter with basic information about the study and the researchers. There was no prior relationship between the interviewer and any of the participants. Recruitment continued until theoretical saturation was reached.

### 2.3 Topics and procedure

The interview guide was developed by WB and EB and sent for feedback to another researcher who had prior experience with interviewing clinical geneticists. Topics of the interviews included the purpose of the informed consent process, the informational needs of patients and how to fulfill those needs, transfer of information prior, during, and after pre‐test counseling, challenges the interviewees experience and their wishes for future policy and practice (see Supporting Information). Interviews were held by WB and took 30 to 45 minutes. Interviews were held in Dutch either over the phone or at the participants' offices. All participants were interviewed once and no other people were present at the interviews. All participants provided oral consent prior to being interviewed.

### 2.4 Analysis

Interviews were audio recorded and transcribed verbatim. Transcripts were deidentified and analyzed using thematic analysis (codebook approach) (Braun & Clarke, 2021) and qualitatively coded with NVIVO 12 software. Main themes were derived from the interview topics and additional themes that emerged during coding were added. The first three interviews were coded independently by WB and EB in order to reach agreement on the list of codes. Coding and analysis continued until no more themes could be identified. The quotes from the interviews presented in this paper were translated to English by WB.

## 3 RESULTS

### 3.1 Research participants

Between October 2019 and January 2020, 14 clinical geneticists from six academic hospitals across the Netherlands participated in the interview study. Participants had the following subspecialisms; neurogenetics (5), cardiogenetics (4), congenital diseases (4), developmental disorders and mental disabilities (3), oncogenetics (2), genetic kidney diseases (2), hearing impairment (2), and immune genetics (1). Most participants had more than one subspecialism. Ten participants were female and four participants were male. The median age was 49 (35–61) and the median years of experience as a clinical geneticist (including training) was 16 (5–27).

### 3.2 Themes

Analysis of respondents' answers to questions posed in the context of the first research question (how do they inform competent adult patients about exome sequencing?) led to the identification of three main themes, namely: essential information, understandability and personalization of information, and support in decision‐making. These three themes are reported first. The second and third research questions (challenges and preferences for the future) are reported next.

#### 3.2.1 Essential information

All clinical geneticists were asked with an open question what they consider essential information that all patients need in order to provide informed consent for the genome‐wide test. Five (groups of) information elements were mentioned by the participants. First, respondents always discussed *the aim, nature, and procedure of the test*. Patients need to know why the test is done, what is being tested, and what the testing process entails for patients (i.e., a blood draw), and when the results are expected. Second, respondents felt that during the informed consent process, *possible outcomes of the test* must be discussed. Most respondents present four types of possible outcomes, namely; 1) a genetic explanation is found for the condition for which the patient seeks consultation, 2) no genetic explanation is found (which does not mean there is no genetic explanation), 3) a Variant of Uncertain Significance (VUS) is found, or 4) an unsolicited finding, unrelated to the present condition of the patient, is found. Apart from the need to inform patients about possible outcomes, all clinical geneticists explicitly mention that informing them about unsolicited findings is essential. One clinical geneticist said about the informed consent process:

“You need to inform patients that [unsolicited findings] can be shocking or [comprise] important information for them. I call it our ‘package leaflet’. Unsolicited findings are an important side effect of genome wide testing” (respondent 2.4).

One clinical geneticist explicitly mentioned carrier status (as a possible unsolicited finding) as essential information.

Third, respondents stressed that in general, it is important for patients to be aware of the (clinical, psychological, and social) *consequences of the test*. The actual content of this topic is dependent on the subspecialism and the patient group of the clinical geneticist. If applicable, more specific consequences are discussed, such as consequences for the treatment of the patient, for reproductive decisions or for family members.

“It is important that [patients] know what is being tested and why it is tested, what the possible outcomes are and what could be the consequences of those outcomes. I think those are the most important topics” (respondent 1.1).

Fourth, respondents discussed the *advantages and disadvantages of the test*. Some clinical geneticists emphasized the importance of this topic for the patient's decision whether or not to take the test. They mentioned that patients need to consider the possible advantages (e.g., the chance of obtaining a clear diagnosis, or the chance that the outcome of the test is useful) and the possible disadvantages (e.g., the chance of VUS and unsolicited findings and the possible distress such results can cause, as well as possible consequences for family members).

“I think you should also talk about the big advantage of the test, versus the possible disadvantage. It sounds very good that this is the best test we have to offer you, but I think it is important that they can weigh the advantages and disadvantages for their own specific situation themselves” (respondent 4.3).

Finally, some clinical geneticists considered *background information* essential, such as what DNA is and how the technique works. Others explicitly considered these elements non‐essential or not relevant for the decision of the patient.

#### 3.2.2 Understandability and personalization

Clinical geneticists were asked how they present information to patients in an understandable way. Eight strategies were mentioned for ensuring that patients understand relevant information and for personalizing the informed consent process. First, respondents kept information short and simple. Some respondents explicitly choose to *keep the information minimal* as they are convinced that this makes information understandable for patients. One of the respondents said:

“I always keep it short while I know others explain more. But I doubt whether people really need [more elaborate information] as maybe you [as a doctor] actually confuse people by providing information that is not very relevant for them, for example what DNA looks like” (respondent 6.2).

Second, some respondents *provide background information and use visuals* to explain factual information about exome sequencing. Some spend time explaining what DNA is or how the test works, for example, by explaining that a gene panel includes 1300 DNA tests in one package. Also, some use or draw pictures to show, for example, what DNA looks like or what genetic variation is. Third, respondents use *examples and analogies* to explain possible outcomes of testing, notably unsolicited findings. Most clinical geneticists use examples of possible unsolicited findings and their “actionability” (i.e., whether the test result provides opportunities for treatment or prevention) to make the information less abstract. Frequently used examples are a genetic predisposition for cancer as an actionable unsolicited finding and dementia as a non‐actionable unsolicited finding. Genetic testing is explained by using analogies such as “finding typographical errors in a book.” For gene panels, the metaphors of a sieve, a filter or colored glasses are used to explain that while all genes are sequenced, not all are analyzed. Several clinical geneticists use the analogy of an x‐ray of the heart, which may show a tumor in the lungs, to explain unsolicited findings:

“And sometimes the parallel of a photograph works well, an x‐ray. You make an x‐ray of the heart, but you also see the lungs and maybe there is a problem with the lungs. People understand that very well” (respondent 4.3).

Fourth, clinical geneticists *encourage patients to ask questions*. Fifth, based on based on the conversation and the questions asked by patients, clinical geneticists estimate how much and which information patients want and are able to receive. Accordingly, they *adapt to the level of (language) comprehension* and to patients' informational needs. The strategy of estimating and adapting to the level of comprehension is used by respondents to make information more understandable for patients, but also to personalize information. How much information a clinical geneticist provides is dependent on how much information an individual patient is estimated to be willing and able to process. Some respondents use a teach‐back method to check whether a patient has understood the information.

“It depends on the educational level of the person in front of you, how thorough you [as a clinical geneticist] explain something. That differs. It's not like telling the exact same story every time. I think I let it depend on at least my estimation on what that person seems to understand and what not” (respondent 3.2).

Sixth, some clinical geneticists go through *the informed consent form or the information brochure* during the counseling session to structure the conversation and to make sure that all essential topics are discussed. Other clinical geneticists only refer to these materials for patients to consult at home or later on. Clinical geneticists also refer to specific webpages of, for example, the hospital or the patient organization, which contain clear and reliable information, as well as to informative online videos of the hospital.

Seventh, respondents take a *stepwise approach to informing patients*. Clinical geneticists try to disclose their information step by step according to the informational needs of patients. By keeping it short at first and providing more detailed information if people ask for it or give the impression that they wish to have more information. It is mentioned that when patients do not appear to need a lot of background information, the clinical geneticist will provide only the information that is considered essential for that individual at that specific moment in time.

“But gradually I taught myself to give the essential information briefly and to‐the‐point […]. And if I have the impression that these people do find it important to know the details, then I elaborate a bit more and I provide them with more detailed information” (respondent 2.5).

Finally, clinical geneticists try to *estimate emotional capacity* by getting a sense of how patients feel about the test that is being offered to them and adapt their counseling to the emotional state of the patient. This may result in postponing or refraining from testing. One respondent said:

“I try to find out what the [patient's] emotional state is. Whether someone will not be able to sleep for six weeks or whether someone will think: well, if something [an incidental finding] is found, maybe I am lucky to know about it in time. This entire spectrum exists and I try to estimate it as well as I can. I also link this to my estimation of the chance of a clear diagnosis. If a person is very anxious and I estimate the chance of a diagnosis from the test to be quite low, I point out that when [the patient is] in doubt, maybe it is wiser to not take the test” (respondent 1.2).

#### 3.2.3 Support in decision‐making

Throughout the interviews, clinical geneticists spoke about the ways they support patients in their decision‐making process. Some clinical geneticists find it important that patients make deliberate and well‐considered decisions, and therefore *encourage patients to consider* whether or not they want to do the test. It was mentioned that when a patient quickly states that he or she wants to know “everything,” the clinical geneticist explicitly stimulates the patient to think about the (dis)advantages of the test, or even to postpone the decision.

“If I have any doubt whether people have understood the information, I offer more time to reflect, so they can think about it. Because some people say yes to everything very quickly, but sometimes I feel that they have not considered it very well. Then I give them the information, tell them to read it well, and then I call them later” (respondent 4.2).

On the contrary, another clinical geneticist mentioned having no problem accepting quick and little‐considered decisions. Also, clinical geneticists mentioned that when a patient is hesitant about testing, they *give the patient more time to consider* and make a second appointment. Clinical geneticists suggest to take time to read the information brochures, watch informative videos online, and discuss the decision with family members. Furthermore, clinical geneticists *discuss patients' motivations* for taking the test. They try to find out at the beginning of the session whether the patient came to see them on their own initiative, or just because their treating physician has referred them. Depending on the situation, they then draw attention to other possibly relevant motivations for testing, which the patient might not yet have thought of, such as consequences for family members or reproductive consequences. In oncology, for example, test results usually have clear consequences for the treatment of the patient, and therefore, the decision to take the test is made quickly. In order to be well prepared, however, respondents mentioned that it is still important to encourage the patient to think about the possible impact of the test results, both for the patient him‐ or herself and for relatives.

Moreover, respondents handled informed consent for gene panels differently from informed consent to open exome sequencing. Several clinical geneticists mentioned that *in counseling for a gene panel, they can be more directive* than in counseling for open exome sequencing, which is more complicated and involves more shared decision‐making. Clinical geneticists propose gene panels to patients as their test of choice, while they present open exome sequencing as an *option* to patients, as something to consider.

“With a panel I am more directive, while with a full exome I maybe leave the choice more to them, whether they wish that at all” (respondent 3.2).

Clinical geneticists emphasized that *expectations management* is important; patients should have correct expectations of the possible test results. They use various strategies to prevent patients from having unrealistic expectations, such as asking patients about their expectations, explaining what the test can and cannot tell them, explicitly mentioning the odds (in percentages) that the test will yield a useful result, and explaining more about the possibility of an unclear result (VUS) when the chances of a useful result are relatively small.

“I always start with: you can't rule out something genetic, you can only prove it if it is there. And I always go back to that. Because especially with a negative result, some of the people will react relieved, that it is not genetic. And then I tell them that we do not know that” (respondent 2.4).

Finally, during the informed consent process, respondents are focused on *recognizing and correcting misconceptions*. Clinical geneticists mentioned that the decision of patients to take or not take the test is sometimes based on misconceptions, which the clinical geneticist first had to recognize and correct. Examples of misconceptions are as follows: the consequences of the test results for health or life insurance, whether the doctor could also test for other diseases that run in the family, whether the test is a complete DNA check, and whether the disease tested for could still be prevented by a healthy life style.

#### 3.2.4 Challenges

Throughout the interviews, clinical geneticists mentioned challenges they come across in the informed consent process. First, genomic tests (e.g., gene panels) are increasingly initiated by medical specialists, other than geneticists, also referred to as mainstreaming. This is done for diagnostic purposes or for the purposes of personalized medicine—that is, for making treatment decisions. Clinical geneticists notice that when patients are referred to them for further counseling in case of an unsolicited finding or an unclear result, they have not always been well enough informed by the medical specialist about the test and the possible consequences. The unsolicited finding or difficult‐to‐interpret result may have caught them by surprise.

“I think in practice, if it does not go through us [but through other, non‐geneticist clinicians], that they [those clinicians] present it as just a test. I think it involves little counseling, to be honest” (respondent 5.1).

Clinical geneticists underscored that patients need proper information on genetic testing before agreeing to undergoing exome sequencing, regardless of who counsels them or the setting in which it is offered to them. They also note a lack of guidance for mainstream medical specialists, as current guidelines for genetic counseling are not multidisciplinary, but apply to clinical geneticists only.

The second category of challenges is about barriers for proper counseling. Language barriers and low educational levels of patients were also mentioned as challenges. Counseling patients with whom it is difficult to communicate, either because of a language barrier or because of a lower level of comprehension, is seen as challenging, as it is difficult to explain the essential information to them. In those cases, clinical geneticists feel that the responsibility for decision‐making lies with them rather than with the patient. In such situations they might only offer a gene panel test and not offer the option of an open exome sequencing test, which they would have if there were no language or comprehension barrier. Lastly, respondents mentioned time pressure as a barrier for proper counseling. In some cases, a lot of topics have to be discussed in one counseling session (anamneses, physical examinations, family tree, information about the proposed test).

#### 3.2.5 Preferences for future policy and practice

In response to the question whether respondents had ideas to improve informed consent practices, some stated that written information should be shorter, simpler, and more effective. They recommend starting from a short and clear message, and providing more extensive information (only) if needed or requested. Second, respondents underscored the importance of recent efforts to develop a uniform nationwide policy for informed consent throughout all hospitals in the Netherlands. All patients should receive the same information and choice options, it was felt, regardless of the hospital they happen to visit. Third, consent should be streamlined and decisions made by patients should be centrally registered. As many patients are not only asked for consent for the genetic test itself, but also, for example, for storage of their samples or data in biobanks and for secondary use of deidentified data for future research, consent should be registered in a well‐organized way, involving less paperwork. Clinical geneticists mentioned approaches like broad consent and dynamic consent as options for future improvement.

## 4 DISCUSSION

This study offers insight in the strategies that clinical geneticists use to inform patients about exome sequencing and to support patients' decision‐making. Some of the clinical geneticists in our study explicitly expect or require a well‐considered decision from patients before ordering the test, while others feel that quicker and less considered decisions may suffice, as long as the patient has understood the information. It could be the case that the former group has a thicker notion of what autonomy entails, namely that an autonomous decision should be authentic, that is, befitting the patient's values and goals. In this view, only a deliberate, well‐considered decision is seen as autonomous, while in other views, also less considered decisions can be autonomous, as long as the patient has the opportunity to consider (Faden & Beauchamp, 1986; White, 2018), and thus *chooses* to decide less deliberately.

Informed consent does not require providing a complete package of information to all patients, nor does it require that all patients understand all “information elements” provided as part of the informed consent process. It serves several functions, including the promotion of trust, which need not rest on understanding of facts (Kraft et al., 2019), also in clinical contexts. Our findings reiterate previous calls for moving away from a focus on the technical aspects of genomic sequencing in traditional information‐laden pre‐test counseling models (Vears et al., 2020; Walser et al., 2017), toward models focused rather on value‐based decision‐making and relational ethics (Samuel et al., 2017).

However, the uncovering and correcting of misunderstandings among patients is an important part of the informed consent process (Bernhardt et al., 2015; Gore et al., 2019). Our study confirms some common misunderstandings with respect to genetic testing, for instance: patients sometimes have overly high expectations of what genomic sequencing can do, as was reported elsewhere (Wynn et al., 2018). Our respondents also observed that patients had misconceptions or inflated fears about the negative consequences testing may have for health or life insurance, which is likewise in line with previous reports (Bernhardt et al., 2015; Tomlinson et al., 2016). While there is a (small) risk that genomic test results may have consequences for insurance, the level of risk differs between countries (Bélisle‐Pipon et al., 2019), and it may be outweighed by the benefits of taking the test. Observers are concerned that in mainstream settings, clinicians may not have sufficient time to attend to important aspects of pre‐test counseling, such as solving misunderstandings (Patch & Middleton, 2018).

Some clinical geneticists in our study mentioned that they sometimes only offer a gene panel test and not the option of an open exome test in cases of language barriers or educational barriers. If they felt the patient failed or would fail to understand relevant information about open exome testing, they were not willing to offer it, as they felt it would be ethically inappropriate to do so. This implies that not all patients are offered open exome tests. While it may seem that this may lead to inequitable access to open exome tests, the data in our study are not sufficient to draw that conclusion. If, for example, a certain patient was only offered a gene panel test, and this test did not yield a satisfying result, this patient might subsequently be offered a follow‐up open exome test after all if the clinical geneticist believed that this would have a better chance of a useful result, and after additional counseling. The possibility of follow‐up steps were not discussed in the interviews. The literature on genetic counseling suggests that directiveness is not necessarily inappropriate in all cases (Biesecker et al., 2019; Vears et al., 2020). When promoting the primary aims of genetic counseling, namely; promoting understanding, facilitating decision making, obtaining patients' informed consent, reducing psychological distress, enhancing perceptions of personal control, and advancing adaptation to health‐threatening information and experiences, clinical geneticists should have space to provide guidance to patients if needed (Biesecker et al., 2019).

Written informed consent materials mostly contain information about the technical background, the procedure and the possible results of exome sequencing. These materials focus rather little on the risks and benefits, or the advantages and disadvantages of exome sequencing, while it is suggested that these topics should be included in all informed consent forms (Ayuso et al., 2013). In this respect, written informed consent materials in the Netherlands are not fully in line with theoretical recommendations. The clinical geneticists in our study, however, mentioned that the advantages and disadvantages of exome sequencing for the patient concerned are usually part of their counseling sessions, and thus conveyed orally. As (dis)advantages are dependent on the patient's situation, it might not be possible to capture these in general terms, in writing. Moreover, the weighing of the advantages and disadvantages for the specific patient is at the center of the patient's decision to take the test. A counseling session is always needed to guide this deliberative part of informed consent.

With patients who have difficulties understanding complex information, information should be reduced to the key messages. The most central messages are, according to the respondents in our study, that exome sequencing can have a wide variety of results and consequences. It may lead to a diagnosis, but it is also possible that no explanation is found. Also, it is possible that the laboratory finds something of which the clinical significance remains unclear, and finally, it is possible that it finds something unexpected (i.e., an unsolicited finding). Layered models for consent have been proposed to keep the provision of information about testing minimal and effective and at the same time to allow for tailoring or personalization of information provision (Bunnik, Janssens, et al., 2013). Layered consent means that information is presented to patients in layers, with the first layer containing key messages, and further layers attuning to the informational needs of patients. Essential, or material information, needs to be provided to all patients (Faden & Beauchamp, 1986). In the ethico‐legal literature this is called the “reasonable person standard of disclosure” (Beauchamp & Childress, 2008), and it would be the first layer of information. The contents of the other layers may be more detailed, and may be extended, depending on what is important to the patient in question. Our study shows that layered consent is also used in practice by some clinical geneticists. Some of the respondents explicitly mentioned how they keep their standard information short, clear and simple for all patients, and provide more detailed information only to those who wish for it.

This interview study has limitations. It might the case that the clinical geneticists who responded to our request for participation, generally put more value on the informed consent process than others, which may have introduced bias. Second, while we consider interviews with professionals to be an adequate method to achieve the aim of this research project, observation of the counseling and informed consent process might have provided additional insight. We did try to organize patient interviews and observations, but given the minimal response we received from clinical geneticists, who perceived them as logistically complicated and too much of a time investment, we resorted to individual interviews with clinical geneticists only. For future research we suggest empirical studies of patients' perspectives on the informed consent process and the use of observation as a research method to examine the counseling and informed consent process.

## 5 CONCLUSION

During informed consent conversations with patients about genomic sequencing, Dutch clinical geneticists typically discuss the following topics: the nature and aim of the test, the possible results (including unsolicited findings and VUS) of the test, and the consequences of those results for the patient and their family members. Some use a layered approach to informed consent, focusing on providing essential information at first, attuning further disclosure of information to the needs of the individual patient, and ensuring that key information is disclosed in a clear and understandable way. Layering information is a useful strategy as it focuses on providing a short and simple general explanation at first. Other topics can be discussed, too, depending on the situation and informational needs of the patient. Layered consent allows clinicians to provide proper pre‐test counseling and informed consent processes, even in mainstream settings, or where time is scarce. When incorporated in guidelines for counseling, the results of this study may help improve informed consent practices for clinical genomic sequencing. Topics we suggest to be described in guidelines are the following: to always have a dialog on what taking a genomic sequencing test means to the patient concerned; to layer information in order to answer to the informational needs of individual patients as best as possible; and to keep written information short and understandable.

## CONFLICT OF INTEREST

Wendy Bos has no conflicts of interest to declare. Eline M. Bunnik has no conflicts of interest to declare.

## ETHICAL COMPLIANCE

The study received a waiver from the Erasmus MC research ethics review committee (MEC‐2018‐1733).

## Supporting information


Supinfo S1

Supinfo S2

## Data Availability

Transcript or audio‐files can be made available to individual researchers upon request but will not be made publicly available to protect the privacy of respondents.
