# Supplementary material for: Informed consent practices for exome sequencing: An interview study with clinical geneticists in the Netherlands
Source: Mol Genet Genomic Med. 2022 Feb 11;10(3):e1882. doi: 10.1002/mgg3.1882 (PMC8922961; doi:10.1002/mgg3.1882)
Supplement: Supplementary file 1 — Supinfo S1 [file MGG3-10-e1882-s002.docx]

**Interview guide**

*Researcher introduces herself and the research project and asks for informed consent (recorded).*

1. As a start, could you shortly explain a little about your specialism and the patient group that you counsel? To which patients do you offer Whole Exome Sequencing (WES)?
2. Could you describe in general terms how a counseling session for WES goes? What do you discuss?
3. What is, according to you, the most important aim of the informed consent process for WES?

*Follow-up question:* is there another important aim?

1. WES is a complicated test with various possible results. A lot of information can be provided about WES. How do you structure this information in a counseling session?

*Follow-up question:* what do you find most complicated about counseling for WES?

1. What is, according to you, the most essential information that patients generally need to decide about doing WES?

*Follow-up question:* every patient had different informational needs. How do you determine those needs and how do you adapt to them?

*Follow-up question:* do you feel that most patients understand the information you provided them with?

1. Where do you find input for your counseling? (e.g. guidelines, literature, collegues, et cetera).
2. Is a counseling session often the same, or different with every patient? What are the differences?
3. Is counseling for a gene panel different from counseling for an open WES?
4. Do you give examples of possible test results, examples of incidental findings, et cetera? If so, which examples do you use?
5. Do you explicitly ask patients if they have any questions? Can you give examples of the questions that patients ask?
6. Do you provide the patient with an information brochure? Before, during the session or afterwards?
7. Do you ask the patient to sign an informed consent form? At what moment?
8. Is your experience that patients have already looked up information about the test beforehand? Or do they obtain information in other ways, prior to the counseling session (for example, from their treating physician)?
9. Do patients sometimes come back to you with questions at a later time? If so, what kind of questions, for example?
10. Do most of the patients you see choose to take the test? Does it happen that people do not want the test? How often? And for what reasons? Do patients sometimes reconsider their decision later on?
11. Are there things in the informed consent process that you would like to change? Do you have recommendations / suggestions for improvement with regard to the written informed consent materials and / or guidelines that you have to work with?
12. Optional: are there any other points that I have not mentioned that you would like to comment on?
